# The general social survey-national death index: an innovative new dataset for the social sciences

**DOI:** 10.1186/1756-0500-4-385

**Published:** 2011-10-06

**Authors:** Peter Muennig, Gretchen Johnson, Jibum Kim, Tom W Smith, Zohn Rosen

**Affiliations:** 1 Department of Health Policy and Management, Mailman School of Public Health, Columbia University, 600 W. 168th Street, 6th Floor, New York, NY 10032, USA; 2 National Opinion Research Center, University of Chicago, 1155 E. 60th St. #270, Chicago IL 60637, USA

## Abstract

**Background:**

Social epidemiology seeks in part to understand how social factors--ideas, beliefs, attitudes, actions, and social connections--influence health. However, national health datasets have not kept up with the evolving needs of this cutting-edge area in public health. Sociological datasets that do contain such information, in turn, provide limited health information.

**Findings:**

Our team has prospectively linked three decades of General Social Survey data to mortality information through 2008 via the National Death Index. In this paper, we describe the sample, the core elements of the dataset, and analytical considerations.

**Conclusions:**

The General Social Survey-National Death Index (GSS-NDI), to be released publicly in October 2011, will help shape the future of social epidemiology and other frontier areas of public health research.

## Background

### Addressing new paradigms in public health

As new knowledge is accumulated, the field of public health periodically undergoes paradigm shifts. For instance, the sanitation and clean water revolution was brought about in part by our understanding of the germ theory[1]. This, augmented by the development of vaccines, antibiotics, and removal of disease vectors, ultimately led to an epidemiologic transition from an environment in which infectious disease took lives at a young age to one in which chronic disease in older age became the major focus[2].

This new paradigm required an entirely new way of thinking about health and disease intervention. This challenge was met with longitudinal studies, such as the Framingham Heart Study, which ultimately showed that smoking, drinking, poor diet, and a sedentary lifestyle were major risk factors for poor health[3]. This realization in part led to the development of large national datasets, such as the National Health Interview Survey. Such surveys not only allow researchers to better understand and track health risks nationwide, but they are also valuable resources for generating and testing the hypotheses that move public health forward. These datasets, have, in turn, helped researchers recognize that social factors--such as income and education--are among the most important determinants of population health[4,5]. This discovery provided a small but important push toward the current public health paradigm shift--that economic characteristics and social environments are key to human health and longevity[5].

However, a dataset has not yet been built that allows researchers to study the relationship between these characteristics and mortality. The General Social Survey-National Death Index (GSS-NDI) is an important first step toward creating a dataset that will help researchers understand how such social environments shape health.

### The evolution of social epidemiology

Social risk factors for poor health, such as poverty, failing schools, community characteristics, and weak social ties, appear to be powerful determinants of one's health and life expectancy[5-12]. Such risks are often called "upstream risks" because they form the source of later health threats, and these later health threats, in turn cascade into further health problems[13]. For example, low educational attainment is associated with behavioral risk factors later in life because of a poor understanding of those risks. Low educational attainment also makes it difficult to obtain a good job that affords health insurance and a home in a low crime neighborhood. These risks can also ripple to further risks "downstream." Exposure to crime, for instance, can lead to psychological stress, which increases allostatic load and may, in turn, lead to heart disease[14]. Stress and crime victimization can also alter one's emotional states (e.g., lead to pessimism or misanthropy), further damaging one's health and overall sense of well-being[15-17]. Psychosocial risk factors have also been linked to immune and endocrine system disruption, putting us at risk for infections like the common cold and leading to premature aging of our cells[14,18,19]. This way, we see that a single event early in life can damage health and well-being over the entire life course, with one event leading to a cascade of other events. The net effect of this cascade is a shorter, less healthy life.

Psychological risks also extend outward into the social spheres around us. Perceived racism may cause health problems among minorities who feel stigmatized,[20,21] but individuals who hold racist beliefs also create distress within the larger communities within which they live, and may therefore be harming themselves and non-minorities within their communities[22]. Even more intriguing, our social networks may not only determine whether we drink or smoke, they may even have a profound effect on the shape and size of our bodies[23,24]. Thus, we might infer that social environments influence the nature of our social ties, which in turn shape our health risks in complex ways.

The idea that social risks cause disease go back well over a hundred years, but has only recently taken root[25]. In the mid-19^th ^century, for instance, Rudolf Virchow posited that poor social policies were a major root cause of diseases such as typhus[26]. However, research on social factors as a cause of disease has been a piecemeal effort. This may be in part because linkages between income and health or education and health were never followed with more powerful survey tools for exploring the social causation of disease.

### The need for a social epidemiology dataset

As they stand, our national health datasets were designed to identify basic correlates of disease, so that these risks can be better described and tracked. But they lack critical information on one's social position, social networks, thoughts, feelings, attitudes, beliefs, and participation in civil society.

If the GSS had been designed as a health dataset from the start it would have had the potential to advance social epidemiologic studies of health disparities beyond mere identification and description to a deeper understanding of the underlying mechanisms. It does contain a few health measures, such as self-rated health and basic information on behavioral risk factors such as smoking (albeit not in all years). However, it was primarily designed to answer traditional questions in sociology and political science. Fortunately, it is possible to link the GSS to prospective mortality data by cause of death for the years 1978 onward via the NDI, so it can be used to answer many new health questions. The GSS-NDI is rich in sociological variables and can provide some of the critical information necessary to better explore the relationship between mortality and social position, beliefs, social cognition, and social relationships.

### Construction and content

The GSS-NDI linked data from 18 waves of the GSS (1978 through 2002) to death certificate data from 1979 through 2008. We have not yet linked more recent years of the GSS to the NDI because very few deaths occur among subjects in the 4- to 6-year period following the survey. It is straightforward and inexpensive to create further linkages as additional mortality data becomes available.

### Sampling

Up through 2006, the GSS sampled only English speaking subjects 18 and over in the non-institutionalized population. Spanish was added as a survey language that year onward, but will not be available in the current GSS-NDI dataset. The GSS employs a multi-stage probability sample. Interviewers conduct face-to-face canvassing after 3 PM on weekdays and on weekends and holidays[27]. Over the 1978-2002 GSS surveys response rates ranged from 70% to 82%. Information on non-respondents is available from the National Opinion Research Center (NORC) at the University of Chicago, host to the GSS. This information can be used to test for non-response bias.

### National Death Index matching

Each subject within the GSS from 1978 through 2002 has a paper record securely stored with identifying information at NORC. To generate a matching file for the NDI, it was necessary to pull paper records for the GSS. Quality control checks included a comparison of names in household enumeration file with names in the questionnaire and a comparison of entered values with GSS public use data. NORC submitted 33,091 records from the GSS to the NDI for linkage. Of these records, 38 were outright rejected and dropped from the dataset because of incomplete or inaccurate information. An additional 223 records were dropped because there were multiple matches between GSS and NDI records. The final sample was therefore 32,830 subjects.

The NDI utilizes a probabilistic matching algorithm to link identifiers from user provided data with death certificate information. This algorithm is designed to maximize the number of correct matches between a survey record and a death certificate, and minimize the number of incorrect matches. The NDI is capable and willing to conduct multiple matches between record files and death certificates. By attempting various permutations of potential errors in a given record file, the probability of a match is increased.

Therefore, prior to submission of GSS records to the NDI, NORC created duplicates of the 660 GSS subject records that had an incomplete birth month, incomplete birth year, or a possible inconsistency in spelling of first or last name of the respondent, yielding a total of 9,824 duplications. These inconsistencies were identified only for those cases in which the paper record was initially entered incorrectly into the computer system (and subsequently caught on the second round of cross-checking), under the assumption that these permutations were common. In cases where the month of birth was missing, NORC created 12 copies of the original record, one with each of the twelve months inserted. Where the age did not match the birth year on the GSS, both birth years were entered.

One issue that affected the match between the GSS identifiers and those in the NDI is that the GSS does not have Social Security numbers for the majority of its subjects. In the GSS, the provision of one's Social Security number was voluntary, and only collected from 1993 onward. Of the GSS records returned as potential death certificate matches, only about 21% had a valid Social Security number.

Roughly 83 to 92 percent of deceased individuals, and 92 to 99 percent of living persons, would be expected to be correctly identified for datasets missing Social Security numbers[28]. The present dataset would be expected to have a higher success rate because Social Security numbers were available for some subjects. This not only improves the likelihood of correct matching for those subjects with Social Security numbers, but also provides an important internal check to help determine the extent to which missing data is or is not influencing the number of successful or incorrect matches.

### Match assessment

Records flagged by the NDI as potential matches were not necessarily interpreted as deaths. Rather, because the NDI utilizes a lenient set of criteria to identify potential matches, these matches are regarded as a set containing both true and false death certificate matches. Of the 22,062 GSS records (or 67% of all records) that the NDI identified as having at least one potential death record match, 5,561 GSS records were matched with a single NDI record. However, 16,501 of the GSS records matched more than one NDI record, with one GSS case being linked to as many as 1,200 NDI records.

To guide the user in assessing the death record matches, the NDI provides a probabilistic matching score. This score was generated by the NDI using previous matching data in which the decedents were known, and provides a reliable way of ascertaining each participant's vital status. Using this algorithm, the NDI generates a suggested vital status (either dead or alive) for each matched NDI record along with the probabilistic matching score.

However, the NDI's matching algorithm uses a weighed set of key identifiers (Social Security number, first and last name, date of birth, gender, race, state of birth, and state of residence) to assign the probabilistic matching score. This procedure disproportionately weighs the number of correct digits in the Social Security number, which was not appropriate for matching GSS records (because many such numbers were missing).

After providing a probabilistic matching score, the NDI produces a class rating that includes five possible classes, dependent on the level of agreement on specific identifiers (Table 1). According to this system, all class 1 matches are considered by the NDI to be an exact match (a death), and all class 5 matches are considered by the NDI to be a false match (alive).

**Table 1:** Definition of Matching Classes from the NDI

Class 1: Exact match on SSN, (all nine digits), first name, middle initial, last names, sex, state of birth, birth month and birth year.
Class 2: Class 2: SSN matches on at least *seven *digits and one or more of the other items from Class 1 may not match. *Note: Some matched cases are moved from Class 2 to Class 5 because of an indication that the reported SSN belongs to the spouse. This includes those cases for which the SSN is known and matches, but the first name and sex do not agree*.
Class 3: SSN unknown but eight or more of first name, middle initial, last name, birth day, birth month, birth year, sex, race, marital status, or state of birth match.
Class 4: Same as Class 3 but less than eight items match.
Class 5: SSN is known but doesn't match. *Note: Some matched cases are moved from Class 5 to Class 3 because of an indication that one of the SSN's (on the user record or on the death certificate) may have been reported incorrectly but a significant number of other data items are in agreement*.

For the GSS-linked records there were no class 1 matches and a limited number of class 2 matches due to the lack of Social Security information and the fact that the GSS did not collect data on participant's state of birth. Subsequently, even for those few records that matched exactly on Social Security number, lack of state of birth information demoted those from class 1 to class 2, and matches that would have met criteria for class 3 were assigned to class 4.

To correct for our missing matching variables, we used a modified cutoff score that lowered the threshold for obtaining a status 1 match (vital status 'deceased') by the exact amount that the NDI gives records matching on seven or eight digits of the Social Security number. Where the GSS records had Social Security number available, the NDI's suggested cutoff score was accepted as accurate.

To identify the correct match, we first selected the match with a combination of the highest class and score provided by the NDI. Of the 22,062 GSS records that the NDI linked with one or more NDI records, we identified 9,285 GSS records (or about 42% of matched records) as having a vital status of deceased. Of these GSS records identified as deaths, the score plus class evaluation flagged 6,504 NDI records (about 70%) as an exact match, but 30.5% of GSS records were linked to more than one NDI record with precisely the same score and class combination. To resolve these multiple matches, we examined the degree to which GSS identifiers agreed with NDI identifiers, and flagged those with the highest number of agreeing identifiers.

The final version of the GSS-NDI dataset contains 32,830 total records, of which 9,271 are deaths. Table 2 shows the sample size and number of subjects identified as deceased for each wave of the GSS-NDI, and Table 3 shows the sample sizes and number of deceased individuals broken down by gender, race, age category and whether the subject was born in a foreign country.

**Table 2:** Sample Size and Mortality in the GSS-NDI by Survey Wave

Wave	N	N Dead	% Dead
1978	1,509	689	45.7
1980	1,274	583	45.8
1982	1,715	746	43.5
1983	1,349	550	40.7
1984	1,411	552	39.1
1985	1,439	632	43.9
1986	1,363	558	40.9
1987	1,725	626	36.9
1988	1,451	513	35.4
1989	1,486	497	33.5
1990	1,346	427	31.7
1991	1,486	454	30.5
1993	1,547	338	21.9
1994	2,949	587	19.9
1996	2,835	478	16.9
1998	2,712	404	14.9
2000	2,650	357	13.5
2002	2,583	280	10.8
All Waves	32,830	9,271	28.2

**Table 3:** Sample Size and Mortality for Selected GSS Subgroups

	Full Sample	Alive	Dead
Number of Subjects	32,830	23,782	9,271
Gender (% Female)	56.7	57.8	54.0
Race			
% White	81.7	81.9	81.2
% Black	14.3	13.4	16.4
% Other	4.0	4.6	2.4
Age at interview			
% 18-35	35.9	44.0	15.1
% 36-65	47.4	47.9	46.2
% 66-90	16.7	8.1	38.6
% Foreign born	7.3	7.8	5.8

### Cause of death linkage

Of the 9,271 GSS records determined to have a vital status of 'deceased', 99.84% were linked to underlying cause of death information. Until the year 1999, the NDI provided users with cause of death using codes from the Ninth Revision of the International Classification of Diseases (ICD-9) and Tenth Revision (ICD-10) codes thereafter. In order to unify the different coding schemes, we collapsed cause of death into 285 mutually-exclusive categories using the single-level Clinical Classification Software (CCS) system.

### Robustness check

Whereas the NDI's method for evaluating matches relied heavily on the Social Security number, our algorithm places emphasis on identifiers used in the match. Our objective was to ensure that our technique was conservative enough to eliminate false positives and provide an accurate record matching. We tested this by utilizing a sub-sample of our matches - the 927 records that were qualified as 'class 2' matches by the NDI's algorithm and thus had a valid Social Security number.

To perform our comparison, we took these matches and modified them to appear as though these records were missing Social Security numbers. This was achieved by reducing the probabilistic score of each match by the Social Security number weight. Next, we re-ran our evaluation method and selected the best match according to the criteria described above. This method identified 784 deaths out of the 927 records that were initially categorized as class 2 matches, proving to be somewhat more stringent than the NDI's evaluation scheme, which designates 860 of the class 2 matches as 'deaths' (a difference of about 8.2%). In effect, this test demonstrates that although the Social Security number provides important information for the linking process, it is not essential for match evaluation. When techniques are undertaken to de-emphasize Social Security number in the match evaluation process, as is the case with our methods, NDI record matches can be assessed so as to maximize sensitivity without sacrificing specificity.

## Utility and Discussion

The GSS-NDI represents a new and essential tool to be used by social epidemiologists. The GSS-NDI will have time trends available for roughly 1000 variables, which will allow researchers to examine relationships between social factors and mortality over time. Because the sample is nationally-representative, repeated variables also open the door to the creation of synthetic cohorts. (E.g., subjects who are age 20-24 in 1980 can be thought of as subjects who are 25-29 in 1985.) Several thousand other variables are available in the GSS-NDI, although many of these were asked during a particular wave of the GSS, and therefore do not have potential for time trend analyses.

In addition, the primary sampling unit information (a de-identified indicator of the geographic location of the interview, which can be employed for participant clustering) can be used for hierarchical linear models. We are currently in the process of setting up a unit that can manage requests for spatial data that cannot be released due to concerns surrounding the possibility of identifying a subject.

One variable, a four-point self-rated health scale (excellent, good, fair, and poor) is available for all years of the GSS-NDI in the public release. This will allow researchers to better probe questions of causality. For example, if we wished to examine the relationship between wealth and mortality, we could limit the analysis to respondents who report excellent or good health at the time of survey. This way, we would be more certain that we were measuring the effect of wealth on illness and not illness on wealth.

We plan on releasing the GSS-NDI to the general public in October of 2011. The dataset will be de-identified, and available for use by researchers everywhere. It has been granted approval from the Institutional Review Board at Columbia University. To ensure that subjects cannot be identified, only the year of the subject's birth and the de-identified primary sampling unit (rather than the subject's city or state of residence) will be available in the public release dataset.

In the meantime, a team of inter-disciplinary researchers is testing the dataset and developing statistical code that can be used as a toolkit for future analyses. Already underway are studies surrounding how changes in discrimination against lesbians and gays may be influencing their health, how the falling academic performance of males is influencing their health, and various research questions surrounding social capital and mortality, variations in mortality with economic cycles, and the underlying nature of the religion-mortality association.

## Conclusions

The newly constructed GSS-NDI dataset was designed to meet the needs of social epidemiologists investigating emerging areas of public health. Researchers who have exhausted the capabilities of our currently available national health datasets ought to find the dataset invaluable in furthering their studies.

## Availability and requirements

The dataset is expected to be publicly available in October 2011 from NORC.

## Competing interests

The authors declare that they have no competing interests.

## Authors' contributions

PM conceived of the paper, wrote the primary text, and contributed to the paper's ongoing development. JK and TS assisted with data preparation and contributed to the development of the paper, GJ conducted the data analyses and contributed to the development of the paper, and ZR helped conceptualize the paper and contributed to its development. All authors read and approved the final manuscript.

## References

[B1] Rosen G A history of public health 1993 Baltimore: Johns Hopkins Univ Press

[B2] Omran AR The epidemiologic transition: a theory of the epidemiology of population change The Milbank Memorial Fund Quarterly 1971 49 4 509 538 5155251

[B3] Krieger N Theories for social epidemiology in the 21st century: an ecosocial perspective Int J Epidemiol 2001 30 4 668 677 10.1093/ije/30.4.668 11511581

[B4] Kitagawa EM Hauser PM Differential mortality in the United States; a study in socioeconomic epidemiology 1973 Cambridge: Harvard Univ. Press

[B5] Muennig P Fiscella K Tancredi D Franks P The relative health burden of selected social and behavioral risk factors in the United States: implications for policy Am J Public Health 2010 100 9 1758 1764 10.2105/AJPH.2009.165019 PMC2920959 20019300

[B6] Woolf SH Johnson RE Fryer GE Rust G Satcher D The health impact of resolving racial disparities: an analysis of US mortality data Am J Public Health 2004 94 12 2078 2081 10.2105/ajph.94.12.2078 PMC1448594 15569956

[B7] Berkman LF Social epidemiology: social determinants of health in the United States: are we losing ground? Annu Rev Public Health 2009 30 27 41 10.1146/annurev.publhealth.031308.100310 19705554

[B8] Kawachi I Subramanian SV Kim D Social Capital and Health 2010 New York: Springer

[B9] Muennig P Jia H Lee R Lubetkin E I think therefore I am: perceived ideal weight as a determinant of health Am J Public Health 2008 98 3 501 506 10.2105/AJPH.2007.114769 PMC2253567 18235062

[B10] Muennig P The social costs of childhood lead exposure in the post-lead regulation era Arch Pediatr Adolesc Med 2009 163 9 844 849 10.1001/archpediatrics.2009.128 19736339

[B11] Muennig P Schweinhart L Montie J Neidell M Effects of a prekindergarten educational intervention on adult health: 37-year follow-up results of a randomized controlled trial Am J Public Health 2009 99 8 1431 1437 10.2105/AJPH.2008.148353 PMC2707464 19542034

[B12] Muennig P Robertson D Johnson G Campbell F Pungello EP Neidell M The effect of an early education program on adult health: the Carolina Abecedarian Project randomized controlled trial Am J Public Health 2011 101 3 512 516 10.2105/AJPH.2010.200063 PMC3036683 21233425

[B13] Gehlert S Sohmer D Sacks T Mininger C McClintock M Olopade O Targeting health disparities: A model linking upstream determinants to downstream interventions Health Affairs 2008 27 2 339 10.1377/hlthaff.27.2.339 PMC2494954 18332488

[B14] McEwen BS Protective and damaging effects of stress mediators N Engl J Med 1998 338 3 171 179 10.1056/NEJM199801153380307 9428819

[B15] Cohen S Doyle WJ Turner RB Alper CM Skoner DP Emotional style and susceptibility to the common cold Psychosom Med 2003 65 4 652 657 10.1097/01.psy.0000077508.57784.da 12883117

[B16] Kubzansky LD Kawachi I Going to the heart of the matter: do negative emotions cause coronary heart disease? J Psychosom Res 2000 48 (4 5):323-337 10.1016/s0022-3999(99)00091-4 10880655

[B17] Yan LL Liu K Matthews KA Daviglus ML Ferguson TF Kiefe CI Psychosocial factors and risk of hypertension: the Coronary Artery Risk Development in Young Adults (CARDIA) study JAMA 2003 290 16 2138 2148 10.1001/jama.290.16.2138 14570949

[B18] Cohen S Psychological stress and susceptibility to upper respiratory infections Am J Respir Crit Care Med 1995 152 4 Pt 2 S53 58 10.1164/ajrccm/152.4_Pt_2.S53 7551414

[B19] Epel ES Blackburn EH Lin J Dhabhar FS Adler NE Morrow JD Cawthon RM Accelerated telomere shortening in response to life stress Proc Natl Acad Sci USA 2004 101 49 17312 17315 10.1073/pnas.0407162101 PMC534658 15574496

[B20] McNeilly MD Robinson EL Anderson NB Pieper CF Shah A Toth PS Martin P Jackson D Saulter TD White C Effects of racist provocation and social support on cardiovascular reactivity in African American women Int J Behav Med 1995 2 4 321 338 10.1207/s15327558ijbm0204_3 16250771

[B21] Steffen PR McNeilly M Anderson N Sherwood A Effects of perceived racism and anger inhibition on ambulatory blood pressure in African Americans Psychosom Med 2003 65 5 746 750 10.1097/01.psy.0000079380.95903.78 14508015

[B22] Kennedy BP Kawachi I Lochner K Jones C Prothrow-Stith D (Dis)respect and black mortality Ethn Dis 1997 7 3 207 214 9467703

[B23] Christakis NA Fowler JH The spread of obesity in a large social network over 32 years N Engl J Med 2007 357 4 370 379 10.1056/NEJMsa066082 17652652

[B24] Christakis NA Fowler JH The collective dynamics of smoking in a large social network N Engl J Med 2008 358 21 2249 2258 10.1056/NEJMsa0706154 PMC2822344 18499567

[B25] Krieger N Historical roots of social epidemiology: socioeconomic gradients in health and contextual analysis Int J Epidemiol 2001 30 4 899 900 10.1093/ije/30.4.899 11511625

[B26] Virchow R Notes on the typhoid epidemic prevailing in Upper Silesia Arch Pathologische Anatomic Physiologic Klinische Medizin 1849 2 143 322

[B27] Smith TW Marsden P Hout M Kim J General social surveys, 1972-2010 NORC: Chicago: Appendix A. Sample design and weighting 2011 http://www.norc.org/NR/rdonlyres/21C53AAC-1267-43B6-A915-A38857DC9D63/1281/AppendixA.pdf Accessed 3/23/2011

[B28] Williams BC Demitrack LB Fries BE The accuracy of the National Death Index when personal identifiers other than Social Security number are used Am J Public Health 1992 82 8 1145 1147 10.2105/ajph.82.8.1145 PMC1695740 1636839

